# Muscle Side Population Cells from Dystrophic or Injured Muscle Adopt a Fibro-Adipogenic Fate

**DOI:** 10.1371/journal.pone.0054553

**Published:** 2013-01-15

**Authors:** Christopher M. Penton, Jennifer M. Thomas-Ahner, Eric K. Johnson, Cynthia McAllister, Federica Montanaro

**Affiliations:** 1 Center for Gene Therapy, The Research Institute at Nationwide Children's Hospital, Columbus, Ohio, United States of America; 2 Biomedical Sciences Graduate Program, The Ohio State University, Columbus, Ohio, United States of America; 3 Comprehensive Cancer Center, The Ohio State University, Columbus, Ohio, United States of America; 4 Ohio State Biochemistry Program, The Ohio State University, Columbus, Ohio, United States of America; 5 Morphology Core, The Research Institute at Nationwide Children's Hospital, Columbus, Ohio, United States of America; 6 Department of Pediatrics, The Ohio State University College of Medicine, Columbus, Ohio, United States of America; University of Minnesota Medical School, United States of America

## Abstract

Muscle side population (SP) cells are rare multipotent stem cells that can participate in myogenesis and muscle regeneration upon transplantation. While they have been primarily studied for the development of cell-based therapies for Duchenne muscular dystrophy, little is known regarding their non-muscle lineage choices or whether the dystrophic muscle environment affects their ability to repair muscle. Unfortunately, the study of muscle SP cells has been challenged by their low abundance and the absence of specific SP cell markers. To address these issues, we developed culture conditions for the propagation and spontaneous multi-lineage differentiation of muscle SP cells. Using this approach, we show that SP cells from wild type muscle robustly differentiate into satellite cells and form myotubes without requiring co-culture with myogenic cells. Furthermore, this myogenic activity is associated with SP cells negative for immune (CD45) and vascular (CD31) markers but positive for Pax7, Sca1, and the mesenchymal progenitor marker PDGFRα. Additionally, our studies revealed that SP cells isolated from dystrophic or cardiotoxin-injured muscle fail to undergo myogenesis. Instead, these SP cells rapidly expand giving rise to fibroblast and adipocyte progenitors (FAPs) and to their differentiated progeny, fibroblasts and adipocytes. Our findings indicate that muscle damage affects the lineage choices of muscle SP cells, promoting their differentiation along fibro-adipogenic lineages while inhibiting myogenesis. These results have implications for a possible role of muscle SP cells in fibrosis and fat deposition in muscular dystrophy. In addition, our studies provide a useful *in vitro* system to analyze SP cell biology in both normal and pathological conditions.

## Introduction

Adult skeletal muscle exhibits a robust regenerative response following injury. Impairment of this response with aging or due to genetic mutations leads to loss of muscle mass and ultimately loss of muscle function. Therefore, intense research efforts are aimed at understanding the cellular and molecular mechanisms that drive muscle regeneration, as they may reveal insights into muscle disease mechanisms.

The primary cellular effector of regeneration is the muscle satellite cell; a stem cell that resides in close apposition with the myofiber, underneath the basal lamina [1]. Satellite cells respond to muscle damage by re-entering the cell cycle to both self-renew and to generate myoblasts that will eventually undergo terminal differentiation and fuse with myofibers to repair damage [2]. Although satellite cells represent the primary source of myogenic cells for regeneration, additional populations of cells have been identified that can undergo myogenic differentiation upon muscle injury [3] and interest has grown towards understanding their roles in the highly coordinated process of muscle repair.

Among these populations are muscle side population (SP) cells. Transplantation studies using gender miss-matched or tagged donor SP cells have revealed that muscle SP cells can participate in muscle regeneration by giving rise to satellite cells [4]–[10]. Importantly, muscle SP cells can engraft into damaged muscles following systemic delivery [4], [6], [7] and they preferentially repopulate the satellite cell niche with the potential for long term muscle regeneration [9]. Therefore, muscle SP cells are being investigated for their potential use in body-wide cell-based therapies for muscle diseases, such as muscular dystrophies where muscle regeneration progressively fails and satellite cells appear to be depleted [11]–[13]. However, recent studies have cast doubt on the ability of muscle SP cells to contribute to myogenesis in injured muscle when they are not manipulated *ex vivo* for transplantation [14]–[16]. These studies do not invalidate the potential usefulness of SP cells in transplantations for cell-based therapies, but they indicate a need to develop tools to better understand the biology of SP cells.

SP cells are isolated by Fluorescence Activated Cell Sorter (FACS) based on their unique ability to efficiently efflux the DNA binding dye Hoechst 33342 [4], [17]. This property is primarily dependent on the activity of the Abcg2 transporter [16], [18]. However, Abcg2 expression is not restricted to SP cells in muscle [9], [16] and not all SP cells express Abcg2 [9], [10]. Indeed, muscle SP cells are heterogeneous with respect to the expression of several markers [5], [10], [17]. The most abundant sub-population (about 80% of the SP fraction in non-injured adult mouse muscle) comprises SP cells associated with blood vessels that express the vascular endothelial marker CD31 [9], [10]. A second sub-population (2% to 10% of total muscle SP) is blood-derived and expresses the immune marker CD45 [19]–[21]. Their number increases in the presence of muscle damage [4], [5], [10], [20]. CD31+ and CD45+ SP sub-populations express high levels of Abcg2 and *in vivo* studies suggest that they might contribute to muscle regeneration by facilitating tissue vascularization and modulating the immune response [16]. Finally, the myogenic activity of muscle SP cells is primarily accounted for by a third sub-population that comprises about 5% of the total SP and does not express CD31 or CD45. This sub-population is referred to as lineage negative SP (Lin- SP) and may express the satellite cell marker Pax7 as well as Abcg2 [9], although these findings are currently controversial [10].

Lin- SP cells are particularly interesting because among all SP sub-populations they have the greatest muscle engraftment and myogenic differentiation potential [10]. Lin- SP cells proliferate in response to acute muscle injury and subsequently preferentially repopulate the satellite cell compartment but also contribute to fully differentiated myofibers [9], [10]. In addition, Lin- SP cells give rise to non-myogenic cells that reside in the interstitium [22]. Interestingly, these non-myogenic cells interact with myogenic cells to enhance their engraftment and promote muscle regeneration. To date, the identity of the non-myogenic cells generated by Lin- SP cells is unknown and it is technically difficult to ascertain *in vivo* due to the lack of a unique Lin- SP marker allowing clear lineage tracing studies and to their very low abundance (Lin- SP cells comprise about 0.1% of muscle mononuclear cells). In addition, no single culture system currently supports the multi-lineage differentiation of muscle SP cells. Instead, differentiation of muscle SP cells into hematopoietic, adipogenic, or osteogenic cells has only been demonstrated with media that contain potent inducers of these lineages [5], [10]. Even their *in vitro* myogenic differentiation is not spontaneous but typically requires co-culture with myogenic cells [5], [10], [23]. To identify the non-myogenic cell types generated by Lin- SP cells in wild type and injured muscle, we developed a culture system that supports their spontaneous multi-lineage differentiation and closely mirrors *in vivo* findings from SP and Lin- SP cell transplantation studies.

## Results

### Muscle SP cells are capable of cell-autonomous myogenic differentiation in vitro

We undertook a screening to identify cell culture conditions that would sustain the propagation of muscle SP cells isolated from wild type murine limb muscles. Similar to prior reports, muscle SP cells could not be maintained on a collagen or gelatin substrate in media that are commonly used for the growth and differentiation of primary muscle cells or of myogenic cell lines [24], [25]. On a Matrigel substrate, a few muscle SP cells adhered but in our hands they did not form muscle as previously reported [26] but gave rise to small colonies of flat cells that were morphologically similar to fibroblasts (data not shown). Even a commercially available medium specifically formulated for skeletal muscle cell growth (Lonza SkGM) did not support muscle SP cells but it did support the growth and differentiation of mouse primary muscle cells (data not shown). We also tested DMEM supplemented with 20% fetal bovine serum and 0.5 nM basic FGF. This formulation is similar to that used by Tanaka et al. [9]. We confirmed that this medium supports the proliferation of SP cells. However, differentiation was not observed as long as bFGF was present in the medium (data not shown).

As part of our screening, we included a medium formulated for the propagation of microvascular endothelial cells (Lonza EGM-2-MV). We tested this medium because the majority of SP cells from wild type muscle express several vascular endothelial markers, they bind the lectin Ulex europaeus agglutinin (UEA), and they incorporate acetylated LDL both *in vitro and in vivo* (Table 1; [10]), indicating that they constitute a sub-population of vascular endothelial cells that are lining blood vessels.

**Table 1:** Muscle SP cells express vascular endothelial cell markers and incorporate Acetylated-LDL in vivo.

% positive cells	CD31	Ly6C	VE- cadherin	UEA	Ac- LDL *in vitro*	Ac- LDL *in vivo*
Muscle SP	90.5±7.1 (n = 12)	75.3±8.5 (n = 4)	89.8±9.2 (n = 5)	78.6± 11.5 (n = 4)	90±7 (n = 5)	70.3±13 (n = 7)

Values represent mean ± standard deviation from the indicated number (n) of biological replicates.

**Figure 1:**
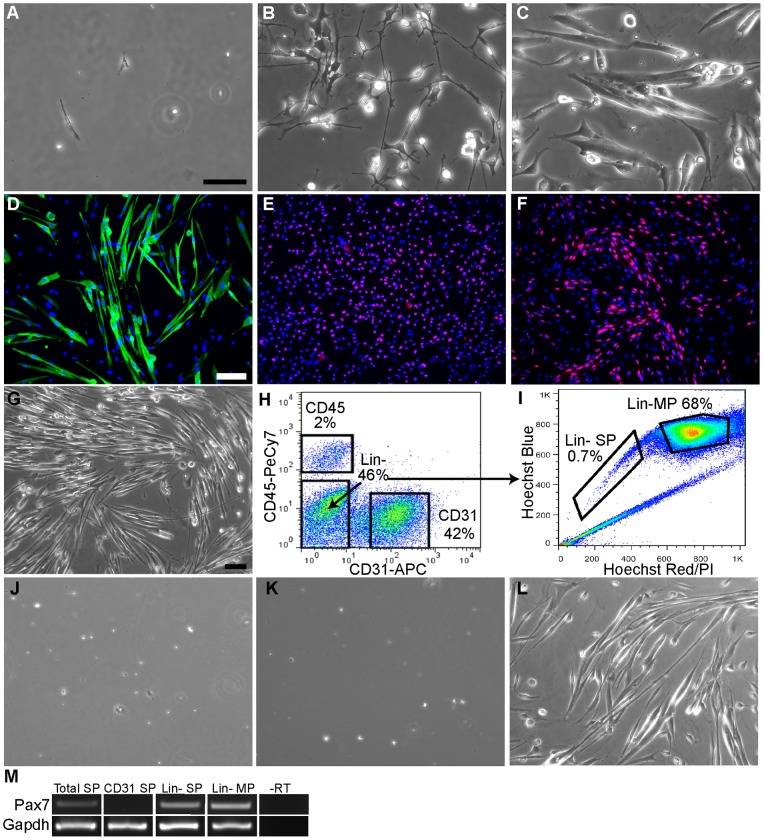
In vitro cell-autonomous myogenic differentiation of muscle SP cells and Lin- SP cells. **A–C**: Phase pictures of wild type muscle SP cells cultured in EGM medium on Matrigel. Cells attached to Matrigel at days 3 (**A**), had significantly proliferated by day 7 (**B**), and differentiated into contracting multinucleated myotubes by day 11 (**C**). Scale bar  = 50 μm. **D–F**: Immunostaining of muscle SP cultures for the myotube marker α-actinin (**D**; green) at day 11; for the myoblast marker myogenin (**E**; green) at day 5; and for the satellite cell marker Pax 7 (F; green) at day 3. Cells were counterstained with DAPI (blue) to visualize nuclei. Scale bar  = 100 μm. **G**: Phase picture of day 11 cultures of primary myogenic cells in EGM medium on a Matrigel substrate showing formation of large numbers of myotubes. **H–I**: Isolation of muscle SP sub-populations by FACS. Total muscle mononuclear cells were labeled with antibodies to CD31 and CD45 and CD31+, CD45+ and Lin- cells were sorted first (**H**). Cells in each fraction were re-analyzed by FACS for incorporation of Hoechst and SP cells were isolated. The SP profile for the Lin- subset of cells in **H** is shown in **I**. MP cells are indicated. **J–L**: Phase pictures of day 11 cultures of CD45+ SP cells (**J**), CD31+ SP cells (**K**), and Lin- SP cells (**L**). Scale bar  = 50 μm. **M**: Pax7 mRNA was detected by RT-PCR in RNA isolated from muscle SP and Lin-SP cells but not CD31+/CD45− SP cells, Lin- MP cells shown in **I** were used as a positive control since they contain satellite cells. RNA was isolated immediately after cell isolation by FACS from wild type muscles.

Interestingly, muscle SP cells cultured in microvascular EGM medium on either a gelatin or Matrigel substrate became adherent within 3 to 4 days and then rapidly proliferated (Figure 1A and B). Because cells cultured on Matrigel appeared healthier and reached confluency faster, Matrigel was used as a substrate for all subsequent studies.

Unexpectedly, within 11 days of plating, muscle SP cells cultured in EGM medium differentiated into multinucleated myotubes that express α-actinin and spontaneously contract (Figure 1C and D). Analysis of cultures at earlier time points revealed that muscle SP cells robustly gave rise to satellite cells (identified by expression of Pax7; Figure 1E) and to committed myoblasts (identified by expression of myogenin; Figure 1F). Terminal differentiation of muscle SP cells into myotubes did not require a switch to a medium with lower levels of serum or mitogens, as is the case for primary muscle cells and myogenic cell lines [27]–[30]. Similarly, the EGM medium was capable of sustaining both the growth and spontaneous differentiation of primary muscle cells without requiring a switch to differentiation conditions (Figure 1G).

Overall these results indicate that the EGM-based *in vitro* system supports the expansion and differentiation of muscle SP cells into satellite cells, myoblasts and ultimately myotubes, thus mirroring *in vivo* results from SP cell transplantations [4]–[8]. Importantly, muscle SP cells expressed their myogenic potential robustly in our *in vitro* system without requiring co-culture with myogenic cells, indicating that they are capable of cell autonomous muscle differentiation.

### The Lin-SP sub-population is responsible for muscle SP myogenesis in vitro

We next asked what SP sub-population (CD31+/CD45−, CD31−/CD45+, or Lin- SP) expanded in our *in vitro* system and differentiated into muscle. We first isolated by FACS three cell fractions: CD45 positive immune cells, CD31 positive vascular endothelial cells and cells negative for either lineage marker (Lin-; Figure 1H). Hoechst incorporation was then visualized for each pre-sorted cellular sub-fraction to further isolate CD31+/CD45− SP, CD31−/CD45+ SP, or Lin- SP cells (Figure 1I and data not shown) and culture them in EGM medium. CD31+/CD45− and CD31−/CD45+ SP cells did not adhere to Matrigel and died after a week in culture (Figure 1J and K). By contrast, Lin- SP cells were indistinguishable from total muscle SP cell cultures generating small branched or spindle shaped cells that readily proliferated and then fused to form myotubes (Figure 1L). Interestingly, only one fifth of Lin- SP cells (500 cells per mm^2^) were needed for successful growth in culture compared to the total SP fraction (2,500 cells per mm^2^). This number agrees with the observation that Lin-SP cells represent about one fifth to one tenth of the total SP cell pool (10%–20%) based on marker expression by FACS (Table 1; [10]). Therefore, the myogenic potential of cultured muscle SP cells can be attributed to the Lin- SP cell fraction.

We next tested for expression of Pax7 transcript in freshly isolated total muscle SP, Lin- SP and CD31+/CD45− SP cells. Pax7 mRNA was detected in total SP and in Lin- SP cells that have myogenic potential, but not in vascular associated CD31+/CD45− SP cells. Therefore the Lin- SP sub-fraction that is myogenic expresses the satellite cell marker Pax7 when freshly isolated from wild type muscle.

### Lin- SP cells are activated in acutely injured and dystrophic muscle

**Figure 2:**
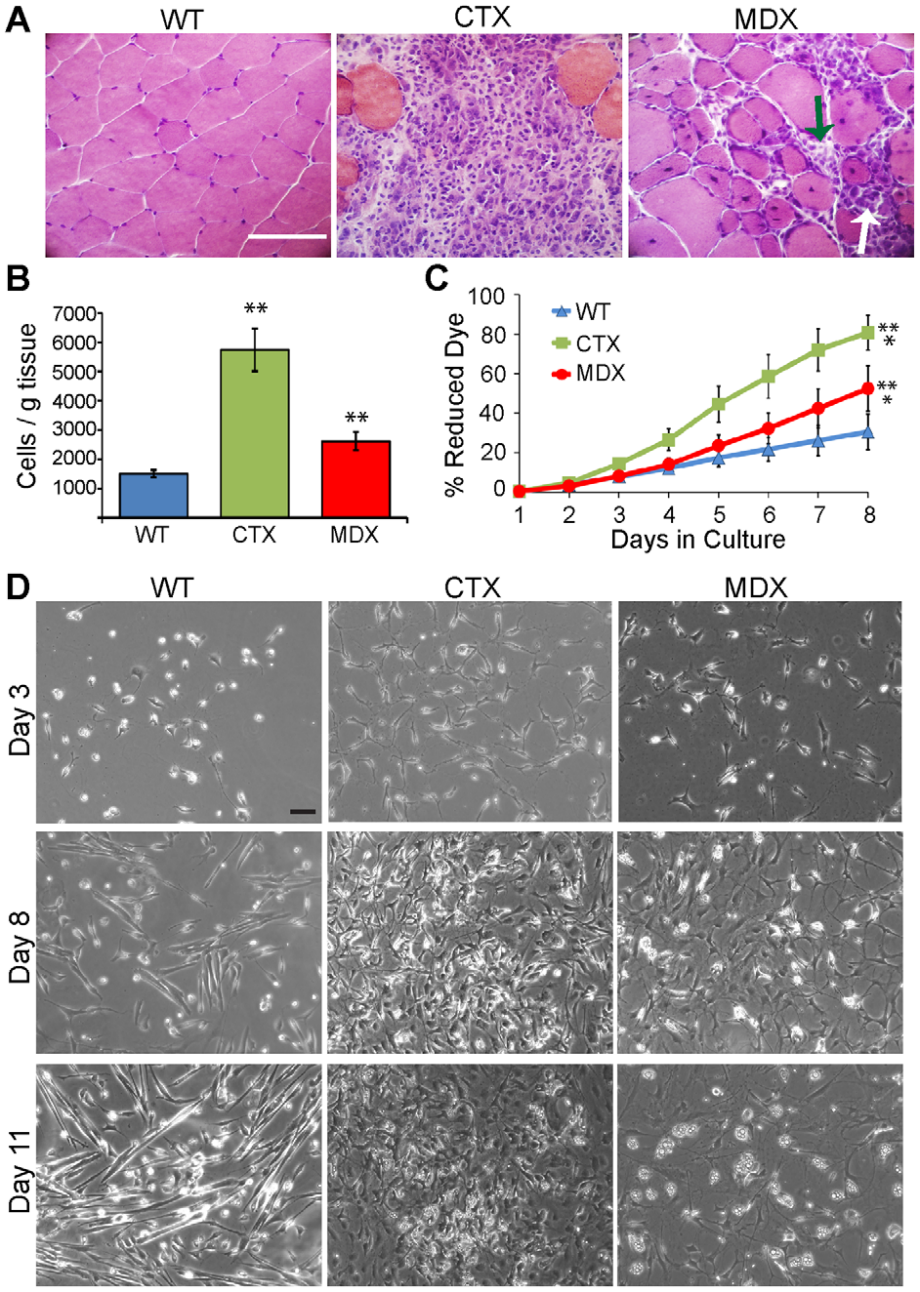
CTX-injury and muscular dystrophy activate Lin- SP cells *in vivo* and alter their *in vitro* proliferation and morphology. **A**: Histological comparison of tissue sections from wild type (WT), CTX-injured (CTX), and *mdx^5cv^* (MDX) tibialis anterior muscle stained with Hematoxylin and Eosin. Wild type muscle shows closely apposed muscle fibers with peripherally located nuclei. CTX-injured muscle at 3 days post-injection has only a few degenerating myofibers surrounded by large numbers of mononuclear cells. Muscle from 8 week old *mdx^5cv^* mice shows areas of active regeneration (white arrow), and areas of muscle degeneration with accumulations of inflammatory cells (green arrow). Scale bar  = 50 μm. **B**: Comparison of the number of Lin-SP cells isolated by FACS per gram of muscle from wild type, CTX-injured and *mdx^5cv^* mice. Asterisks indicate a significant difference (p<0.01, Student's t-test) from wild type muscle. **C**: Quantification of *in vitro* Lin- SP cell proliferation using the Alamar Blue vital dye assay. Asterisks indicate a significant difference (p<0.001, linear regression analysis) from wild type muscle. **C**: Phase pictures of wild type, CTX and *mdx^5cv^* Lin- SP cells at days 3, 8 and 11 in culture showing differences in cell adhesion, proliferation and morphology. Scale bar  = 50 μm.

Prior *in vivo* transplantation studies have suggested that acute muscle injury induced by cardiotoxin (CTX) increases the non-myogenic differentiation of Lin- SP cells [22]. Since we are interested in identifying this non-myogenic cell type, we isolated and cultured Lin- SP cells from CTX injured muscle. Cells were isolated at day 3 post-CTX injection for consistency with the prior *in vivo* study [22]. Day 3 also represents the peak of Lin- SP cell expansion following CTX injury [10]. We further extended our study to include Lin- SP cells isolated from dystrophic muscles of *mdx^5cv^* mice, a model for Duchenne Muscular Dystrophy (DMD), where skeletal muscles undergo chronic muscle damage [31]–[33]. Unlike CTX injured muscle that goes through a cycle of synchronized myofiber degeneration followed by regeneration, muscles of 8 week old *mdx^5cv^* mice show focal areas of degeneration and regeneration that co-exist within the same muscle (Figure 2A). We therefore expected Lin- SP cells to show different responses to these two very different modalities of muscle damage.

We first determined whether chronic muscle injury in dystrophic muscles also activates Lin- SP cells similar to CTX injury. We quantified the total number of Lin-SP cells per gram of muscle tissue in 8 week old *mdx^5cv^* mice and compared it to non injured and CTX injured wild type mice. We confirmed that CTX injury induces a significant increase (about 5-fold) in Lin- SP cells compared to non injured wild type muscle (Figure 2B). In addition, we found that the number of Lin- SP cells was also significantly increased in *mdx^5cv^* muscles by about 2-fold (Figure 2B), indicating that Lin- SP cells are activated in dystrophic muscle. When cells were cultured in our *in vitro* system, *mdx^5cv^* Lin- SP cells and especially CTX Lin- SP cells showed significantly faster proliferation rates compared to Lin- SP cells isolated from non injured wild type muscle (Figure 2C). *In vitro* proliferation rates (Figure 2C) closely matched *in vivo* cell quantification results (Figure 2B), with Lin- SP cells from dystrophic muscle showing slightly lower proliferation rates than Lin- SP cells from CTX injured muscle. Differences in proliferation were also evident by visual observation of cells in culture. Wild type Lin- SP cells only became adherent after 3 days in culture and progressively increased in number from day 4 to day 11 without reaching confluence (Figure 2D, WT column). By contrast, Lin- SP cells from CTX-injured and *mdx^5cv^* muscles adhered to the culture substrate within 24 hours and rapidly began to proliferate reaching confluence by day 8 for CTX Lin- SP cells and by day 11 for *mdx^5cv^* cultures (Figure 2D, CTX and MDX columns respectively).

These results indicate that Lin- SP cells are activated *in vivo* in both CTX-injured and dystrophic muscle. This activation results in faster adhesion to the substrate and a significant increase in proliferation rate *in vitro*.

### The Lin- SP cell fraction from CTX-injured and *mdx^5cv^* muscles does not form muscle in vitro

Visual observation of the cultures at day 11 revealed a lack of multinucleated contracting myotubes in cultures of Lin- SP cells from CTX-injured and *mdx^5cv^* muscles (Figure 2D). Instead the predominant cell types generated by Lin- SP cells from damaged muscle were branched cells that appeared dark under phase, flat fibroblast-like cells, and small angular cells filled with lipid droplets (Figure 2D). These cell types were present but rare in cultures of wild type Lin- SP cells where spindle shaped cells and myotubes were the predominant cell types, and myotubes were readily detectable by day 8 (Figure 2D).

**Figure 3:**
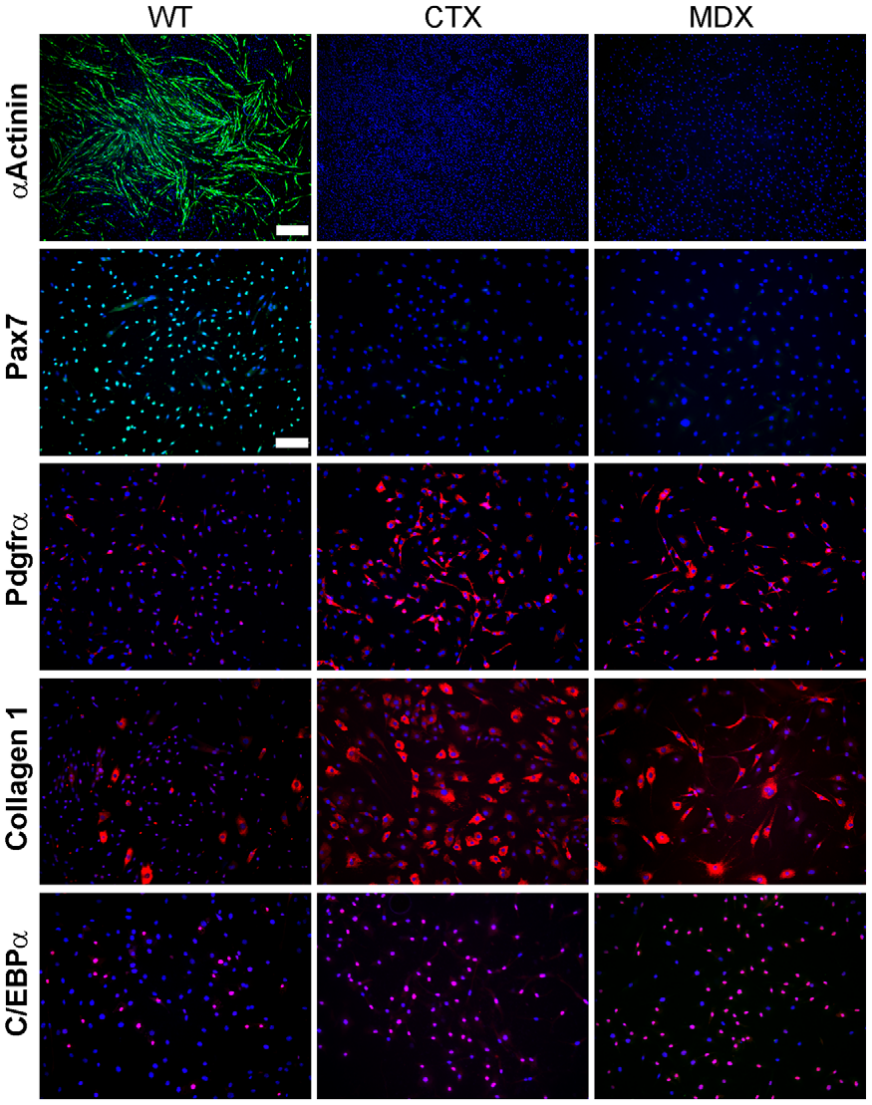
Muscle damage abolishes *in vitro* myogenesis of Lin- SP cells and favors their differentiation into FAPs, fibroblasts and adipocytes. Cultures of Lin- SP cells isolated from wild type (WT) CTX-injured (CTX), or *mdx^5cv^* mice were immunolabelled with antibodies to the myogenic (green) or mesenchymal (red) markers indicated. Labeling for Pax7 (satellite cells), PDGFRα (FAPs) and Collagen 1 (fibroblasts) was done on day 7 cultures. Labeling for α-actinin (myotubes) and C/ebpα (adipocytes) was performed at day 11. Scale bar for α-actinin pictures is 500 μm. Scale bar shown in WT Pax7 picture applies to all other pictures and is 100 μm.

To confirm the lack of myogenic differentiation of CTX and *mdx^5cv^* Lin-SP cells, day 11 cultures were immunolabelled for α-actinin, a protein highly expressed in myotubes. No α-actinin labeling was observed in three independent biological replicates of CTX or *mdx^5cv^* Lin-SP cultures, while myotubes were covering the entire surface of wild type Lin-SP cultures (Figure 3). We then analyzed early cultures of Lin- SP cells for the presence of Pax7-positive satellite cells. At day 7, half the cells in cultures of wild type Lin-SP cells were Pax7-positive satellite cells (Table 2; Figure 3). By contrast, no Pax7 staining was observed in cultures of CTX or *mdx^5cv^* Lin-SP cells at day 7 (Table 2; Figure 3) or at earlier time points (days 1 and 5; data not shown). Therefore, lack of myogenesis in cultures of Lin- SP cells from CTX-injured or dystrophic muscle appears to be due to a lack of satellite cells in these cultures.

**Table 2:** In vitro lineage choices of Lin-SP cells isolated from wild type, mdx5cv, or cardiotoxin-injured muscle.

	Days in culture	Day 7	Day 7	Day 11	Day 14
	Cell type	Myogenic	FAPs	Fibroblast	Fat
	Marker	Pax7	PDGFRα	Collagen I	CEBPα
% cells positive	Wild type	52±6	8±5	12±3	22±3
	*Mdx^5cv^*	0	67±9	60±7	55±1
	Cardiotoxin	0	48±8	40±2	53±4

Values represent mean ± standard deviation from 3 biological replicates.

**Figure 4:**
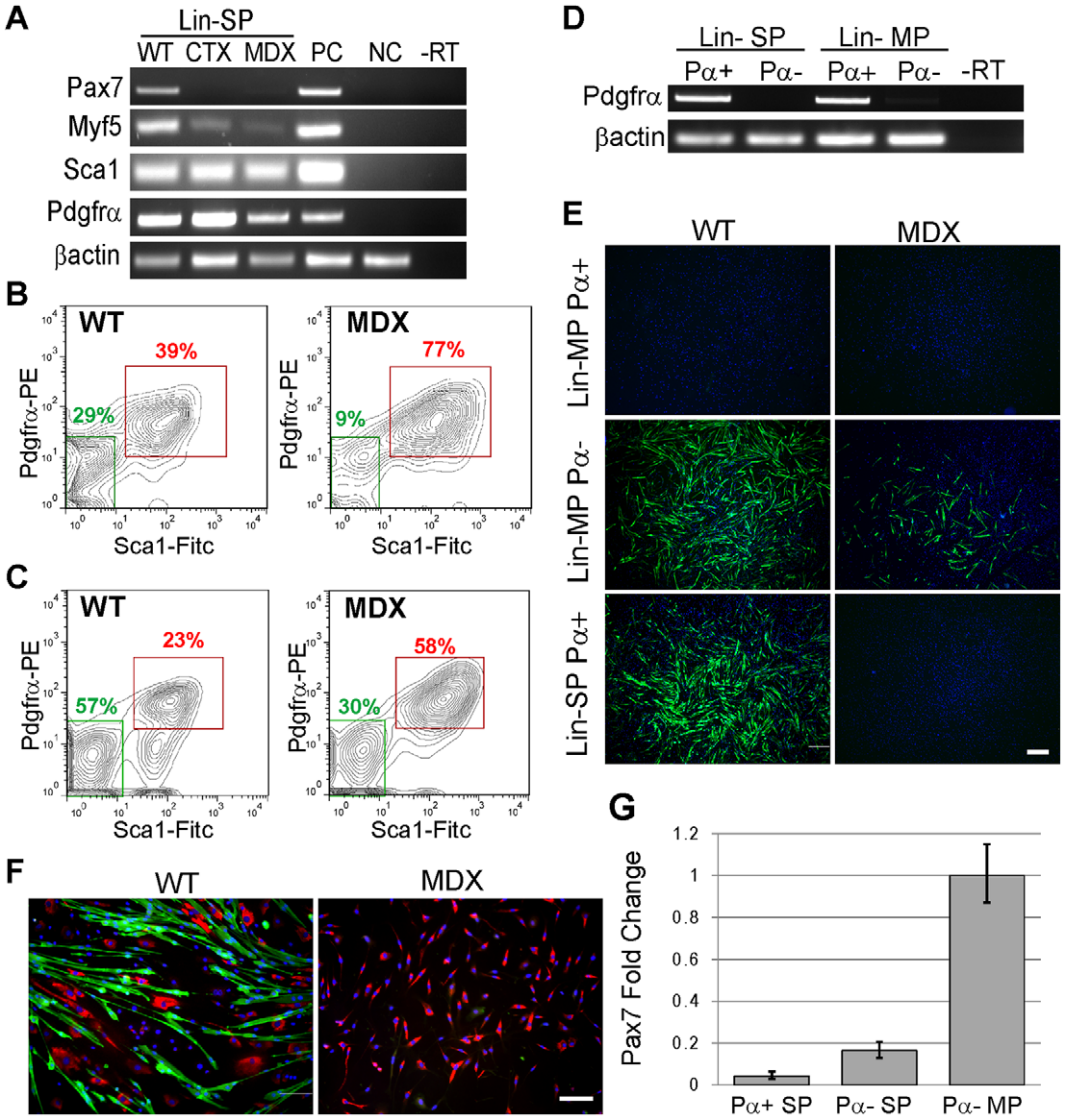
Freshly isolated Lin-SP cells express FAPs surface markers but are capable of myogenic differentiation. **A**: RT-PCR analysis of freshly isolated Lin- SP cells from wild type (WT) and *mdx^5cv^* (MDX) muscle for myogenic markers (Pax7 and Myf5) and FAPs markers (PDGFRα and Sca1). Positive controls (PC) are sorted Sca1-positive cells for Sca1 and Lin- MP cells for Pax7, Myf5 and PDGFRα. Negative controls (NC) are sorted Sca1-negative cells for Sca1 and CD45-positive MP cells for Pax7, Myf5 and PDGFRα. **B, C**: FACS analysis of PDGFRα and Sca1 protein expression in Lin- SP cells (**B**) and Lin- MP cells (**C**) from wild type (WT) and *mdx^5cv^* (MDX) muscle. Percentages of cells double positive (red) and double negative (green) for PDGFRα and Sca1 are shown. **D**: Confirmation by RT-PCR for PDGFRα expression in Lin- SP and Lin- MP cells sorted into PDGFRα-positive (Pα+) and PDGFRα-negative (Pα−) sub-fractions. **E**: *In vitro* myogenic differentiation of Lin- SP and MP cells sorted based on PDGFRα (Pα) expression. Cells were fixed after 14 days in culture and immunolabelled for α-actinin (green) to reveal myotubes. Cultures were counterstained with DAPI (blue) to visualize nuclei. Lin- MP Pα+ cells correspond to the previously characterized FAPs. Lin- MP Pα− cells are enriched in myogenic cells and also contain fibroblasts. Cultured wild type Lin- MP Pα− cells had 2,261 myotubes while cultured *mdx^5cv^* Lin- MP Pα− cells had only 541 myotubes. Lin- SP Pα− cells did not survive in culture and are not shown. Scale bar  = 400 μm. **E**: Cultures of Lin- SP Pα− cells were double labeled with antibodies to α-actinin (green) and collagen I (red) to visualize myotubes and fibroblasts, respectively. Cultures of Lin- SP Pα− cells from dystrophic muscle (MDX) do not contain myotubes but give rise to fibroblasts. Scale bar  = 100 μm. **G**. Quantitative RT-PCR for Pax7 expression in Pdgfrα+ and Pdgfrα− Lin-SP cells, and Pdgfrα− Lin-MP cells. Data is presented as means +/− s.d. from 3 technical replicates.

To further probe this question, we analyzed expression of Pax7 in freshly isolated Lin- SP cells, prior to any culture. As shown in Figure 4A, Pax7 mRNA was not detected in freshly sorted Lin- SP cells from CTX and *mdx^5cv^* muscles. This result was confirmed by quantitative RT-PCR analysis where Pax7 mRNA was detected in wild type Lin- SP cells but was undetectable in CTX and *mdx^5cv^* Lin-SP cells (data not shown). Of note, Pax7 expression in wild type Lin-SP cells was 2.7-fold lower than in a cell fraction enriched for satellite cells (the main population cell fraction that is negative for CD31, CD45, Sca1, and PDGFRα). These data show that Pax7 expression is lost in the Lin-SP cell fraction in response to muscle damage.

To determine whether other markers of satellite cells are expressed by Lin-SP cells and might be similarly affected by muscle damage, we analyzed expression of the early myogenic regulatory factor Myf5, and two cell surface markers expressed by satellite cells: integrin α7 and SM/C-2.6 [34], [35]. RT-PCR analysis of Myf5 mRNA revealed that this transcription factor is present in freshly isolated CTX and *mdx^5cv^* Lin-SP cells, albeit its levels of expression are decreased compared to wild type Lin-SP cells (Figure 4A). Expression of integrin α7 and SM/C-2.6 was assayed by FACS in freshly isolated Lin-SP cells. In wild type muscle, 12% of Lin-SP cells were double positive for integrin α7 and SM/C-2.6. In *mdx^5cv^* muscle, Lin-SP cells double positive for integrin α7 and SM/C-2.6 were still present but their proportion was decreased to 6%. In addition, we found Lin-SP cells that were positive for one satellite cell marker but not the other. Lin-SP cells expressing only the integrin α7 marker represented about 45% of the total Lin-SP cell fraction. Lin-SP cells positive only for the SM/C-2.6 marker represented about 1% of the total Lin-SP cell population. Interestingly, the proportion of integrin α7 or SM/C-2.6 single positive Lin-SP cells did not change between wild type and *mdx^5cv^* muscle.

Taken together, these results indicate that both acute (CTX injury) and chronic, focal muscle damage (*mdx^5cv^* muscle) lead to a loss of *in vitro* myogenic differentiation of Lin- SP cells. This is accompanied by a loss of expression of Pax7 and a decrease in Myf5 expression, as well as a decrease in the proportion of Lin-SP that express the satellite cell markers integrin α7 and SM/C-2.6.

### Muscle damage promotes *in vitro* formation of fibro-adipogenic precursor cells by Lin-SP cells

We next sought to identify the non-muscle cells generated by CTX and *mdx^5cv^* Lin-SP cells. *In vivo* studies have shown that upon transplantation Lin- SP cells isolated from injured muscle give rise to interstitial cells that facilitate myogenesis [22]. The ability to support myogenesis and the interstitial location are reminiscent of the recently identified fibroblast and adipocyte precursors (FAPs) [36]–[38]. FAPs can be identified based on expression of the platelet derived growth factor receptor alpha (PDGFRα), lack of Pax7 expression, and the ability to spontaneously give rise to both fibroblasts and adipocytes when cultured. We first performed immunostaining for PDGFRα in early (day 7) cultures of Lin- SP cells. In cultures of wild type Lin-SP cells PDGFRα was expressed in only a few cells (8.5% of total; Figure 3). By contrast, 48% and 67% of cells expressed PDGFRα in cultures of CTX and *mdx^5cv^* Lin-SP cells, respectively. Furthermore, PDGFRα was never co-expressed with Pax7 (data not shown), in agreement with reports that FAPs are not myogenic [36]–[38]. We next tested for the presence of fibroblasts and adipocytes in later cultures (day 11 and day 14, respectively) of Lin- SP cells. Fibroblasts were identified by immunolabeling for Collagen I, while adipocytes were recognized by immunolabeling for CEBP/α (Figure 3). As shown in Table 2, at day 11, fibroblasts were 3-fold and 5-fold more abundant in CTX and *mdx^5cv^* cultures repsectively compared to wild type. This was accompanied at day 14 by an over 2-fold greater number of adipocytes in both CTX and *mdx^5cv^* cultures compared to wild type (Table 2). Importantly, our culture medium does not contain factors that induce adipogenesis.

Taken together, these results indicate that both acute muscle damage and muscular dystrophy support the differentiation of Lin- SP cells to a fibro-adipogenic lineage *in vitro*. Furthermore, our *in vitro* system suggests that cells within the Lin- SP cell fraction give rise to the recently identified FAPs or contain a small subset of FAPs.

### Freshly isolated Lin-SP cells express FAPs surface markers but are capable of myogenic differentiation

We next asked whether freshly isolated Lin- SP cells expressed two markers of FAPs, namely PDGFRα and Sca1 [38]. Transcripts for both PDGFRα and Sca1 were readily detected by RT-PCR in both wild type and *mdx^5cv^* Lin-SP cells right after isolation by FACS (Figure 4A). To assay for protein expression, freshly isolated Lin- SP cells were double labeled with antibodies to PDGFRα and Sca1 and analyzed by FACS. As shown in Figure 4B, Lin- SP cells are heterogeneous with about 40% of wild type Lin- SP cells expressing both PDGFRα and Sca1. The number of PDGFRα and Sca1 double positive Lin- SP cells increased to 77% in *mdx^5cv^* muscle (Figure 4B), suggesting an expansion of this sub-population in dystrophic muscle.

We next tested which sub-population of wild type Lin- SP cells was responsible for myogenic differentiation *in vitro*. Since PDGFRα positive FAPs do not give rise to myogenic cells and since satellite cells do not typically express PDGFRα and Sca1, we hypothesized that PDGFRα positive Lin- SP cells would give rise to FAP cells, while PDGFRα negative Lin- SP cells would differentiate into myogenic cells. As control, we isolated PDGFRα positive Lin- main population (MP) cells that correspond to previously characterized FAPs, and PDGFRα negative Lin- MP cells that are enriched in myogenic cells (Figure 4C). Of note, FACS analysis indicated that PDGFRα positive Lin- MP cells (FAPs) were more abundant in *mdx^5cv^* muscle compared to wild type muscle. Purity of sorted cell populations was confirmed by RT-PCR analysis for PDGFRα in all 4 sorted cell fractions (Figure 4D). As expected, PDGFRα− Lin- MP cells that are enriched for myogenic cells gave rise to myotubes while no myogenic differentiation was observed in cultures of PDGFRα+ Lin- MP cells (FAPs) from either wild type or dystrophic muscle (Figure 4E). Interestingly, we observed about 4-fold less myotube formation in cultures of PDGFRα− Lin- MP cells from dystrophic muscle compared to wild type (Figure 4E). Contrary to our expectations, we found that PDGFRα+ Lin- SP cells from wild type muscle readily formed myotubes (Figure 4E) indicating that unlike FAPs these cells have myogenic potential. PDGFRα− Lin- SP cells did not survive in culture and could therefore not be assessed for their myogenic potential. Importantly, PDGFRα+ Lin- SP cells from *mdx^5cv^* muscle did not give rise to myogenic lineages (Figure 4E) but readily formed fibroblasts (Figure 4F) and adipocytes (not shown), indicating that the myogenic potential of the PDGFRα+ Lin- SP cell fraction is impaired in dystrophic muscle.

To further explore the unexpected myogenic potential of PDGFRα+ Lin- SP cells, we asked whether these cells expressed satellite cell markers. Quantitative RT-PCR analysis revealed expression of Pax7 in freshly isolated wild type PDGFRα+ Lin- SP cells as well as PDGFRα− Lin- SP cells (Figure 4G). Pax7 expression in these populations was low compared to PDGFRα− Sca1− Lin- MP cells that are enriched in satellite cells. Pax7 expression was not detected in PDGFRα+ Sca1+ Lin- MP cells that correspond to FAPs (data not shown). We next analyzed by FACS the expression of the satellite cell markers integrin α7 and SM/C-2.6 in PDGFRα+ Sca1+ Lin- SP cells from both wild type and *mdx^5cv^* muscle. We found that about 70% and 60% of PDGFRα+ Sca1+ Lin- SP cells from wild type and *mdx^5cv^* muscle respectively are double positive for both integrin α7 and SM/C-2.6.

These results indicate that the myogenic differentiation potential within the Lin-SP cell fraction co-segregates with cells expressing markers typically associated with FAPs (PDGFRα and Sca1) and with satellite cells (Pax7, Myf5, integrin α7 and SM/C-2.6), suggesting that these Lin-SP cells are distinct from both FAPs and satellite cells. Furthermore, among these markers, only Pax7 expression becomes undetectable in dystrophic muscle, and appears to correlate with a loss of *in vitro* myogenic differentiation potential.

## Discussion

Currently, the study of muscle SP cells is complicated by the lack of specific markers, their low abundance, their heterogeneity and the lack of a culture system that supports their multi-lineage differentiation. Here we have identified culture conditions that promote the expansion *and* the multi-lineage (mesenchymal and myogenic) differentiation of the Lin- SP cell fraction. Our EGM-based culture conditions offer several advantages. First, different conditions are not required for myogenic growth and differentiation. Second, these conditions support multiple primary cell types within muscle (myogenic cells, FAPs, fibroblasts and adipocytes) rendering them particularly useful for the study of lineage choices. Third, they closely mirror *in vivo* findings on the response of SP cells to muscle injury with regards to their cell autonomous differentiation into satellite cells, myoblasts and myotubes [4]–[10], their high myogenic potential [9], [10], their increased proliferation in response to injury [9], [10], and their exclusive mesenchymal differentiation when isolated from CTX-injured muscle [22]. Therefore, our culture conditions should prove extremely useful as a complement to *in vivo* experiments. Finally, the EGM medium can support the survival and differentiation of low numbers of SP cells. This allowed us to further sub-fractionate the Lin-SP cell population and to determine that the PDGFRα+, Sca1+, Lin-SP sub-fraction contains cells that can give rise to myogenic and mesenchymal progenitors.

One limitation of this culture system is that it cannot support the clonal growth of Lin-SP cells. At densities below 500 cells/cm^2^, Lin-SP cell viability was compromised and surviving cells adopted a fibroblast-like morphology and did not proliferate. Although we made several attempts at co-culturing single Lin-SP cells expressing green fluorescent protein (GFP) or LacZ with non-marked primary muscle cells, we could not test multi-lineage differentiation. Therefore, at this time we cannot determine whether the PDGFRα+, Sca1+, Lin-SP sub-fraction contains cells with a dual mesenchymal and myogenic differentiation potential, or whether there are separate precursors for each lineage.

Although muscle SP cells and in particular Lin- SP cells give rise to myogenic cells both *in vivo* upon transplantation and *in vitro* [4]–[10], their participation in muscle repair *in situ* remains unclear. Two recent studies have suggested that non-satellite cell populations, including SP cells, do not normally contribute to myogenesis following injury [14], [15]. This conclusion is based on the assumption that only satellite cells express Pax7. However, we show here that SP cells and specifically Lin-SP cells also express Pax7. This is in agreement with prior studies in mouse [9], [39] and human [40] muscle SP cells. Therefore further studies are needed to assess the contribution of Lin-SP cells to muscle regeneration and their relationship to satellite cells. Here, we have shown that a subset of freshly isolated Lin-SP cells shares with satellite cells expression of Pax7, Myf5, integrin α7 and SM/C-2.6. However, the myogenic fraction of Lin-SP cells also uniformly expresses high levels of Sca1 and PDGFRα, two markers not present on satellite cells [41]–[44]. Similarly, a prior study also found that myogenic cells within the Lin-SP occupy a typical satellite cell position at the muscle fiber but express a mix of satellite cell (Pax7, syndecan 4) and non-satellite cell (Abcg2) markers [9]. Taken together these data suggest that myogenic Lin-SP cells may represent a sub-population within the satellite cell pool. These two cell types may therefore be difficult to tease apart. Interestingly, Abcg2 knock-out mice show impaired regeneration with a 30% decrease in satellite cells after a single round of muscle degeneration/regeneration [16]. Importantly, proliferation and differentiation of satellite cells is not affected by loss of Abcg2 expression, indicating that muscle regeneration is affected independently of the canonical Abcg2-negative satellite cell [16]. While these findings support a role for Lin- SP cells in muscle regeneration and satellite cell replenishment, lineage tracing of Abcg2 expressing cells yielded conflicting results [16]. This may be due to the very low level of Abcg2 expression in resting Lin-SP cells [10] that may inefficiently activate the genetic tracing system. As a result, the question of the level of contribution of Lin- SP cells to the satellite cell compartment (and other mesenchymal cell lineages) remains unresolved and awaits the identification of a marker that is strongly expressed by Lin- SP cells and can differentiate them from other cell types.

One of our most interesting findings is that Lin-SP cells express PDGFRα and Sca1, two markers previously associated with FAPs but not myogenic cells [36]–[38]. Culture of Lin-MP cells in our system agrees with previous studies [37], showing that PDGFRα+ cells generate fibroblasts and adipocytes, while PDGFRα− Lin-MP cells are highly enriched for myogenic cells. However, cultures of PDGFRα+ Lin-SP cells yielded highly myogenic colonies when isolated from wild type muscle. These results confirm that the majority of cells within muscle that express PDGFRα are non-myogenic. However, they further reveal that Lin-SP cells represent a unique sub-population of PDGFRα+ cells that have myogenic potential. Future *in vivo* experiments are required in order to determine whether PDGFRα+ Lin- SP cells contribute to muscle regeneration *in vivo*.

Our culture system provides a useful tool to study the effects of the muscle environment on myogenesis in Lin-SP cells. In particular, we have made the important discovery that myogenic differentiation of Lin- SP cells is lost following muscle damage, either acute or chronic. It is unlikely that these results are an artifact of our *in vitro* system because Lin- SP cells isolated from CTX-injured muscle lose their myogenic potential *in vivo* following transplantation and give rise to mesenchymal cells that support myoblast engraftment [22], a property also attributed to FAPs [38]. Overall these results support a model where following muscle damage Lin-SP cells may contribute to an expansion of the population of FAPs that promote the differentiation of myogenic cells. The loss of myogenic potential in Lin-SP cells from CTX-injured or dystrophic muscle can be interpreted in at least two different ways. The first scenario assumes that distinct cell populations within the Lin-SP are responsible for mesenchymal and myogenic differentiation. Loss of Pax7 expression in Lin-SP cells from damaged muscle would be interpreted as a loss of the myogenic fraction from the Lin-SP. This could arise from Pax7+ Lin-SP cells being recruited to participate in muscle repair and differentiate into myogenic cells that no longer efflux Hoechst. The second scenario assumes that cells with myogenic potential are still present in the Lin-SP fraction of damaged muscle but they have lost Pax7 expression and cannot differentiate along a myogenic lineage. Our finding that some markers of satellite cells, namely Myf5, integrin α7 and SM/C-2.6 are still expressed by Lin-SP cells from CTX-injured or dystrophic muscle suggests that cells with myogenic potential may still be present. Interestingly, unfractionated SP cells isolated from Pax7 null mice show a 2-fold decrease in myogenic differentiation but not a complete loss of myogenic potential when co-cultured with myogenic cells [5]. These findings suggest that either a SP cell sub-population distinct from Lin-SP is capable of Pax7-independent myogenic differentiation, or that multiple mechanisms in addition to Pax7 down-regulation are responsible for loss of myogenic potential in Lin-SP cells following muscle damage.

Overall, our culture system reveals a change operated by the dystrophic muscle environment upon Lin-SP cells with potential implications for their use in cell-based treatments of DMD. It will be important to determine whether the presence of chronic muscle damage promotes a continuous production of FAPs, fibroblasts and ultimately adipocytes by Lin-SP cells. Interestingly, *mdx^5cv^* Lin-SP cells show a strong bias for the formation of fibroblasts compared to CTX Lin- SP cell cultures (60% versus 39.5%). It is therefore possible that a sustained production of fibroblasts by Lin- SP cells may over the long term significantly contribute to the progressive fibrosis observed in dystrophic mice [45], [46]. It will therefore be important to determine how *in vivo* ablation of Lin- SP cells, once unique markers are identified, impacts disease progression in dystrophic muscles. In the meantime, our *in vitro* system may prove very useful in the identification of factors within dystrophic and damaged muscle that dictate the fate and behavior of Lin-SP cells.

## Materials and Methods

### Ethics statement

This study was carried out in strict accordance with the recommendations in the Guide for the Care and Use of Laboratory Animals of the National Institutes of Health. All animal protocols were approved by the Animal Care and Use Committee of Nationwide Children's Hospital.

### Animals

Eight to twelve week old female wild type C57BL/6J mice (Jackson Laboratories; Stock 000664) and dystrophin-deficient B6Ros.Cg-Dmd*mdx^5cv^* mice (*mdx^5cv^*; a gift of Louis Kunkel, Harvard Medical School, Boston, MA) were used for all experiments. *Mdx^5cv^* mice have a mutation in exon 10 of the DMD gene that disrupts dystrophin expression and they were originally purchased from Jackson Laboratories (Stock 002379). All mice were bred in house.

### Cardiotoxin injury

Acute muscle damage was induced by intramuscular injection of CTX (Sigma, St. Louis, MO) in wild type C57BL/6 mice. CTX (10 μM in sterile PBS) was unilaterally injected into the tibialis anterior (25 μl), quadriceps femoris (50 μl), and gastrocnemius (25 μl) muscles under anesthesia. Mice were sacrificed for cell isolation or histological analysis 3 days post-injection.

### Cell Isolations

For all SP cell isolations, muscles from 6 to 8 mice were pooled to obtain enough cells for culture or analysis. Muscle cells were isolated from hindlimb skeletal muscles as previously described [17]. Briefly, muscles were trimmed of fat and tendons and finely minced. The minced muscle was digested for up to 30 minutes at 37°C in 1.2 U/ml Dispase (Worthington Biochemicals) and 5 mg/ml Collagenase Type IV (Worthington Biochemicals) in phosphate buffered saline (PBS) containing 2.5 mM CaCl_2_. The enzymes were inactivated with DMEM/10% FBS, and the digest passed through 70 μm and 40 μm cell strainers. Cell suspensions were overlaid on Horse Serum (Gibco) and centrifuged at 160 xg for 10 min without brakes to remove debris.

For general isolation of myogenic cells, the pre-plating technique was used. Briefly, dissociated cells were centrifuged at 450 xg and resuspended in complete EGM2-MV medium (Lonza) without hydrocortisone. Cells were transferred to a 10cm tissue culture dish (BD Falcon) and incubated for 1 hour at 37°C, 5% CO_2_ to allow fibroblasts to adhere. Non adherent cells were removed and re-plated on a fresh 10 cm dish and incubated an additional hour at 37°C, 5% CO_2_ to remove residual fibroblasts. Non-adherent myogenic cells were transferred to a Matrigel coated dish and cultured for 11 days prior to fixation for immunolabeling.

For isolation of SP cells our previously described protocol was followed [17]. Cells were resuspended at 10^6^ cells/ml in PBS containing 0.5% (w/v) bovine serum albumin (Sigma; PBS/BSA) and labeled with 12.5 ug/ml Hoechst 33342 (Sigma) in the presence or absence of 100 mM verapamil (Sigma) for 1 hour at 37°C. Cells were washed in PBS/BSA and counterstained with propidium iodide (Fisher Scientific) to label and exclude dead cells. Cells were visualized and sorted on a FACSVantage DIVA (BD Biosciences) cell sorter using our previously described configuration settings [17]. Cells were collected in culture medium.

For isolation of SP and MP cell sub-fractions, cells were resuspended at 2×10^6^ cells per 100 μl after Hoechst staining and pre-incubated on ice for 10 min in FC block (BD Biosciences). Cells were then labeled with the following primary antibodies for 15 min on ice: CD31-APC (MEC 13.3, BD Biosciences), CD45-PeCy7 (clone 30-F11, BD Biosciences), Ly-6A/E (Sca-1)-FITC (clone E13-167.7, BD Biosciences), and PDGFRα-PE (clone Apa5, Abcam,). Samples were counterstained with propidium iodide (Fisher Scientific) to label and exclude dead cells. The cell sorter was setup to first visualize propidium iodide, CD31-APC and CD45-PeCy7. Live cells (propidium iodide negative) were sorted for each fraction (CD31+/CD45−, CD31−/CD45+ and CD31−/CD45−) using a 4-way sort head. Cells were collected on ice in PBS/BSA and immediately re-analyzed by FACS to visualize Hoechst for isolation of SP and MP cells. In some experiments, Sca1-FITC and PDGFRα-PE were co-visualized with Hoechst to further sub-fractionate Lin- SP and MP cells. For cell culture experiments, sorted cells were directly collected in culture medium. For RNA isolation, cells were collected in Trizol-LS (Invitrogen). From tissue pooled from 6 to 8 mice we routinely obtained 10,000 to 20,000 Lin-SP cells and 5,000 to 10,000 Lin- Pdgfrα+/− SP cells.

### Quantification of Lin- SP cells by FACS

For each biological replicate, hind limb muscles from 3 wild type, CTX-injected or *mdx^5cv^* mice were pooled and weighed prior to cell isolation. The entire cell suspension was labeled for Hoechst, CD31-APC, CD45-PeCy7 and propidium iodide as described above. Lin- SP cells were sorted and automatically counted during collection. The number of cells sorted was divided by the initial muscle weight. Values from 4 to 6 independent biological replicates were used to derive means and standard deviations. Comparisons between WT, CTX, and MDX were performed using a one way ANOVA followed by a Bonferroni pairwise comparison. Significance threshold was set at p<0.05.

### Analysis of vascular marker expression

For cell surface marker analysis, muscle cells were isolated and stained with Hoechst as described above. Cells were resuspended in PBS/BSA at 2×10^6^ cells per 100 μl and 100 μl aliquots were prepared for single antibody or lectin staining. Samples were pre-incubated on ice for 10 min in FC block (BD Biosciences) and then labeled with the following primary antibodies for 15 min on ice: CD31-FITC (clone MEC 13.3, BD Biosciences), VE-Cadherin (clone 11D4.1, BD Biosciences), Ulex europaeus agglutinin I (UEA)-biotin (Vector Labs), and Ly6C-biotin (clone AL-21, BD Biosciences). Cells were rinsed, resuspended in 100 μl PBS/BSA, and the appropriate samples were incubated with Goat anti Rat IgG-FITC secondary antibody (BD Biosciences) or with Streptavidin-FITC (BD Biosciences) for 10 min on ice. Samples were rinsed and counterstained with propidium iodide prior to FACS analysis.

To assay for Ac-LDL uptake *in vivo*, 100 μg Ac-LDL conjugated to Alexa488 was injected in a total volume of 100 μl into the tail vein of wild type mice. Animals were sacrificed 16 hours later and muscles were harvested for cell isolation. Cells were stained with Hoechst and then propidium iodide to eliminate dead cells from analysis. Alexa 488 fluorescence was co-visualized with Hoechst by FACS to quantify the number of live SP cells that had incorporated Ac-LDL. For some experiments, samples were also labeled with an antibody for CD31 directly conjugated to APC (BD Biosciences). In these experiments, all SP cells that had incorporated Ac-LDL *in vivo* were also positive for CD31.

For *ex-vivo* Ac-LDL uptake, cells were stained with Hoechst then incubated for 10 min in ice cold PBS/BSA containing 15 μg/ml Ac-LDL conjugated to Alexa 488. Cells were counterstained with propidium iodide and then analyzed by FACS.

### Cell Culture

All cells were cultured in a 37°C incubator with 5% CO_2_ in complete EGM-2 MV medium (Lonza) without hydrocortisone. Cells were plated in 12 well tissue culture treated plates coated with Matrigel (BD Biosciences) diluted 1∶1 in complete EGM-2 MV medium. Cell density at plating was 2,500 cells per cm^2^ (10,000 cells per well) for total SP cells and 500 cells per cm^2^ (2,000 cells per well) for SP and MP sub-fractionated cells. This resulted in about 4 to 6 cultures to be established per cell isolation from muscle pooled from 6 to 8 mice. Half of the medium was replenished every 4 days.

### Immunocytochemistry and cell quantifications

Cells were fixed in 4% paraformaldehyde for 15 minutes, permeabilized in 0.1% Triton-X100 for 10 min, incubated first for 1 hour at room temperature in blocking solution (PBS with 10% horse serum), and then overnight at 4°C with primary antibodies diluted in blocking solution. Primary antibodies are anti-Pax7 (DSHB), anti-myogenin (clone F5D, DAKO), anti-αactinin (Sigma), anti-Collagen 1 (Cedarlane), anti-PDGFRα (clone APA5, BD Biosciences) and anti-C/EBPα (Santa Cruz). Cells were incubated with Alexa488 or Texas Red conjugated secondary antibodies (Jackson Immunoresearch) for 1 hour at room temperature followed by incubation with DAPI (Invitrogen) to counterstain nuclei. Coverslips were mounted with n-propyl gallate (Sigma) mounting media. To measure the percentage of cells positive for each marker, 8 randomly selected fields were photographed per well. Images were taken from two to three independent experiments. The percentage of positive cells for each differentiation marker was determined by dividing the number of positively stained cells by the total number of nuclei as determined by DAPI counterstain. Comparisons between groups were performed using a one way ANOVA followed by Bonferroni pairwise comparisons. Results were considered significant if p<0.05.

### Alamar Blue Cell Proliferation Assay

Cells were seeded at a density of 1000 cells per well in a 96 well plate coated with Matrigel (BD Biosciences) containing 180 μl EGM-2 MV medium (Lonza). Twenty-four hours after plating, 20 μl Alamar Blue (Invitrogen) reagent was added to each well. Absorbance measurements were taken at 570 nm and 600 nm on a SpectraMax M2 (Molecular Devices) microplate reader and recorded using Softmax Pro software. Readings were recorded once per day from the same wells until 8 days of culture. Proliferation calculations were performed to manufacturer specifications. Linear regression was performed on each group and pairwise comparisons were performed by comparing slopes using ANOVA and Bonferroni correction. Comparisons were considered significant if p<0.05.

### Reverse transcription PCR

Cells were sorted directly into Trizol LS (Invitrogen) and RNA was isolated according to manufacturer specifications. Isolated RNA was further purified using the RNeasy Micro Kit (Qiagen) and quantified on a Picochip. First strand cDNA was synthesized using Superscript III (Invitrogen) from 20ng of input RNA using oligo dT primers. Primer sequences used for RT-PCR are: *Sca1*: Forward-5′- TGGATTCTCAAACAAGGAAAGTAA AGA -3′, Reverse-5′- ACCCAGGATCTCCATACTTTCAATA -3′, *Pdgfrα*: Forward- 5′-GACGAGTGTCCTTCGCCAAAGTG-3′, Reverse-5′-CAAAATCCGACCAAGCACGAGG-3′, *Pax7*: Forward- 5′ CCCAACAGGTTTTCCCAACTG-3′, Reverse-5′- CGGCCTTCTTCTAGGTTCTGCT-3′, *Myf5*: Forward-5′- TTAGCAAACCATGAACACGAAACA-3′, Reverse-5′- AAGGGGGCTTCATTTACCAGG-3′, *Gapdh*: Forward-5′- CACGGCAAATTCAACGGCACAGTCAAGG-3′, Reverse-5′- GTTCACACCCATCACAAACATGG-3′, and βactin: Forward-5′-ATGGAGGGGAATACAGCCC-3′, Reverse- 5′-TTCTTTGCAGCTCCTTGCGTT-3′.

Quantitative RT-PCR was performed on an Applied Biosystems 7500 using Sybr Green Master Mix (Fermentas). Gene expression fold change was determined by the delta-delta Ct method using TATA binding protein (TBP) as the housekeeping gene. Primer sequences for quantitative RT-PCR include Pax7 as previously mentioned, and *TBP*: Forward-5′- CCGTGAATCTTGGCTGTAAACTTG-3′, Reverse-5′- CAACGCAGTTGTCCGTGGCTCTCT-3′.
